# Multiparametric MRI-based radiomics and deep learning for differentiating uterine serous carcinoma from endometrioid carcinoma: a multicenter retrospective study

**DOI:** 10.3389/fonc.2025.1655384

**Published:** 2025-10-08

**Authors:** Yi Shen, Liping Liu, Shuang Ma, Xiaohua Ban, Shaoxian Chen, Zhuozhi Dai, Shaofan Lin, Kainan Huang, Xiaohui Duan, Daiying Lin

**Affiliations:** ^1^ Department of Radiology, Shantou Central Hospital, Shantou, China; ^2^ Department of Radiology, Cancer Hospital of Shantou University Medical College, Shantou, Guangdong, China; ^3^ Department of Radiation Oncology, Sun Yat-sen Memorial Hospital, Sun Yat-sen University, Guangzhou, Guangdong, China; ^4^ Department of Radiology, State Key Laboratory of Oncology in South China, Collaborative Innovation Center for Cancer Medicine, Sun Yat-Sen University Cancer Center, Guangzhou, Guangdong, China; ^5^ Department of Radiology, Sun Yat-sen Memorial Hospital, Sun Yat-sen University, Guangzhou, Guangdong, China; ^6^ Guangdong Provincial Key Laboratory of Malignant Tumor Epigenetics and Gene Regulation, Medical Research Center, Sun Yat-Sen Memorial Hospital, Sun Yat-Sen University, Guangzhou, Guangdong, China

**Keywords:** magnetic resonance imaging, radiomics, deep learning, uterine serous carcinoma, endometrial cancer

## Abstract

**Background:**

Uterine serous carcinoma (USC) and endometrioid endometrial carcinoma (EEC) are distinct subtypes of endometrial cancer with markedly different prognoses and management strategies. Accurate preoperative differentiation between USC and EEC is of great significance for tailoring surgical planning and adjuvant therapy.

**Purpose:**

To develop and validate a multiparametric MRI-based radiomics and deep learning (DL) model for preoperative distinguishing USC from EEC.

**Methods:**

A total of 210 patients (68 USCs and 142 EECs) from four hospitals who underwent preoperative MRI were enrolled in this retrospective study. Features from radiomics and deep learning were extracted using T2-weighted imaging (T2WI), diffusion-weighted imaging (DWI), and contrast enhanced MRI (CE-MRI). The least absolute shrinkage and selection operator (LASSO) analysis was employed to identify the most valuable features. Clinical-radiological characteristics, radiomics and DL features were constructed using a support vector machine (SVM) algorithm. The models were evaluated using receiver operating characteristic (ROC) and decision curve analysis (DCA).

**Results:**

The all-combined model of clinical-radiological characteristics, radiomics and DL features showed better discrimination ability than either alone. The all-combined model demonstrated superior classification performance, achieving an AUC of 0.957 (95% CI: 0.904–1.000) on the internal-testing set and an AUC of 0.880 (95% CI: 0.800–0.961) on the external-testing set. The DLR model demonstrated superior predictive performance compared to the clinical-radiological model, although the differences were not statistically significant in both the internal-testing set (AUC = 0.908 vs. 0.861, *p* = 0.504) and the external-testing set (AUC = 0.767 vs. 0.700, *p* = 0.499). The DCA revealed that the all-combined model illustrated the best overall net benefit in clinical application.

**Conclusion:**

The integrated model, combining multiparametric MRI-based radiomics, deep learning features, and clinical-radiological characteristics, may be utilized for the preoperative differentiation of USC from EEC.

## Introduction

In 2020, endometrial cancer (EC) ranked as the sixth most prevalent cancer among women worldwide, with 417,000 new cases diagnosed (1). Endometrioid carcinoma (EEC) represents the predominant histological subtype of EC, comprising 85-90% of cases. EEC is linked to a reduced risk of progression and a favorable prognosis, especially in low-grade cases (2). Uterine serous carcinoma (USC), the second most prevalent type of EC, constitutes only 5% to 10% of EC cases but accounts for 40% of deaths related to EC (3–6). Patients with USC often exhibit lymph vascular space invasion, nodal involvement, and microscopic peritoneal spread, even in early-stage disease with limited myometrial invasion (3, 7). This leads to a 2.5-fold higher risk of being diagnosed with stage III or IV disease compared to those with EEC (46% in USC vs. 20% in EEC) (7). Surgery is crucial for treating EC, with USC requiring more extensive resection than EEC. Pelvic and paraaortic lymphadenectomy, peritoneal biopsies are recommended for early-stage USC (8).

Currently, the preoperative distinction between USC and EEC relies heavily on invasive procedures such as endometrial biopsy or dilation and curettage (D&C). However, these invasive techniques are susceptible to sampling error in the presence of tumor heterogeneity, not infrequently leading to discordance between preoperative and final postoperative histology (9, 10). For instance, in a large series, nearly one-third of tumors initially diagnosed as low-grade endometrioid carcinoma were upgraded or reclassified as high-grade carcinoma upon examination of the hysterectomy specimen (10). This diagnostic inaccuracy can lead to suboptimal surgical planning. Therefore, a non-invasive method capable of providing a holistic assessment of the entire tumor is highly desirable to complement biopsy findings.

Magnetic resonance imaging (MRI) has been widely used in the diagnosis and differential diagnosis of EC (11–16). A recent study has highlighted the unique MRI characteristics associated with USC, notably heterogeneous signal intensity suggestive of peritoneal dissemination and the presence of abnormal ascites, serving as distinguishing features from EEC (11). Furthermore, imaging parameters derived from diffusion-weighted imaging (DWI), dynamic contrast-enhanced (DCE) MRI, and amide proton transfer (APT) imaging have improved diagnostic accuracy and facilitated the differentiation of endometrial carcinoma subtypes (13–16). However, due to the rarity of USC and consequent limited sample sizes, its preoperative radiological characteristics are not well-defined, and the diagnostic performance of conventional MRI interpretation remains variable and suboptimal, with area under the curve (AUC) values ranging from 0.62 to 0.826 (13, 16).

Radiomics extracts high-throughput features from traditional images and capturing intratumoral heterogeneity that is easily missed by blind biopsies (17). Meanwhile, deep learning (DL) has demonstrated superior performance in image analysis tasks by automatically learning intricate patterns from data (18, 19). These techniques have been increasingly applied in EC for preoperative prediction of high-grade tumors, lymph node metastasis, lymphvascular space invasion, cervical stromal invasion, and deep myometrial invasion (20–27). However, two critical gaps persist in the literatures. First, while previous studies have focused on predicting tumor grade (20, 26) or broadly differentiating type II from type I EC (25), the specific discrimination between USC and EEC—a distinction with significant therapeutic implications—has not been systematically explored using an integrated radiomics and DL approach complemented by clinical-radiological data. Second, most of these previous existing models are derived from single-center cohorts and lack robust external validation, limiting their generalizability.

Therefore, this study aimed to develop and validate, for the first time, a multicenter-integrated model utilizing multiparametric MRI-based clinical, radiomics, and deep learning features for the preoperative differentiation of USC from EEC.

## Materials and methods

### Patients

This retrospective study was approved by the Ethics Committees of the respective institutions, with informed consent waived due to its retrospective nature. Prior to analysis, all patient data was deidentified to ensure the confidentiality and anonymity of personal information.

**Figure 1:**
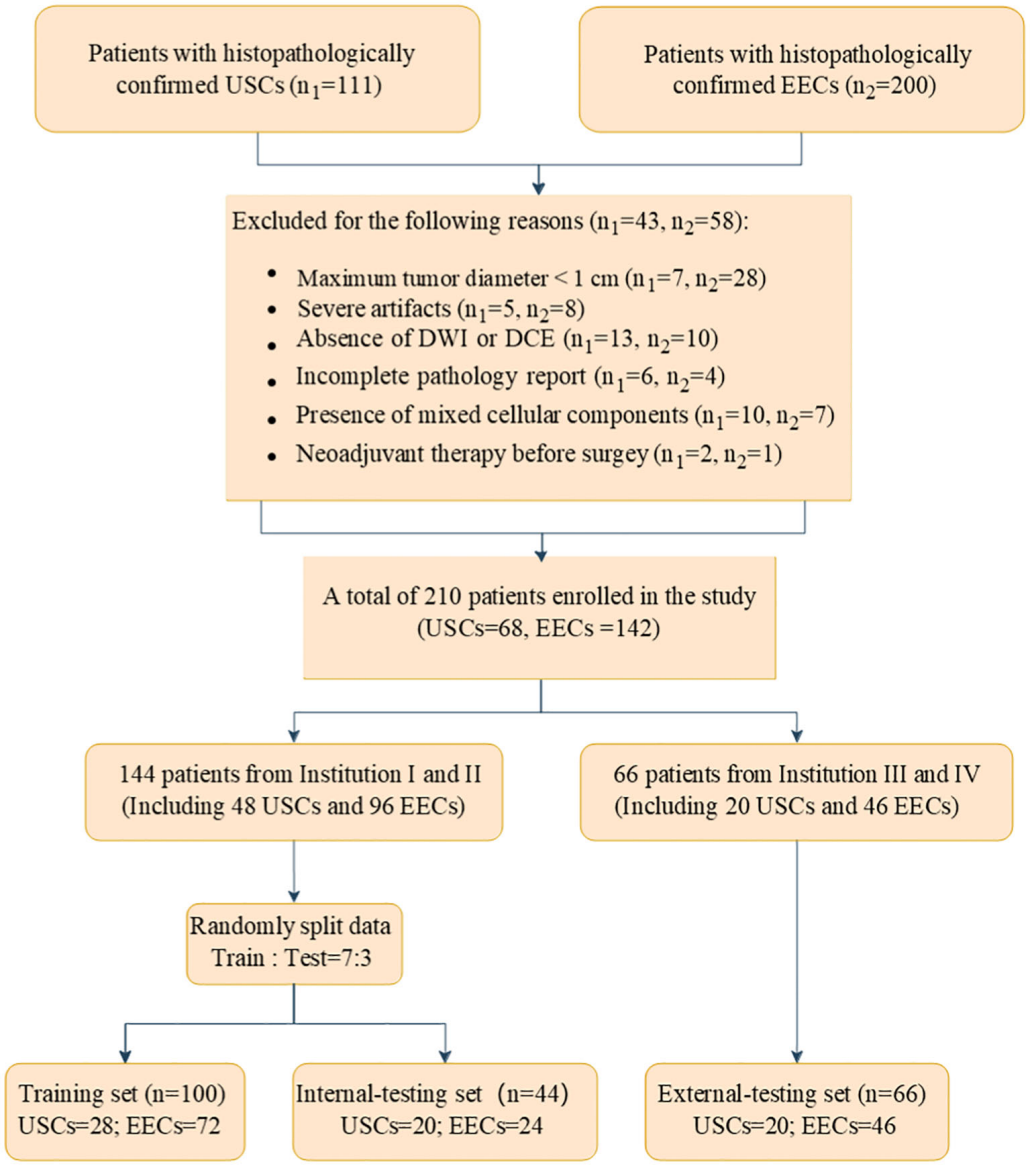
Flowchart of patient recruitment.

We identified a cohort of 311 patients from four medical centers who underwent gynecological surgery, including 111 with USC and 200 with EEC. The participating centers were as follows: Shantou Central Hospital (Institution I), Sun Yat-Sen Memorial Hospital (Institution II), Sun Yat-Sen University Cancer Center (Institution III), and Cancer Hospital of Shantou University Medical College (Institution IV). The specific data collection timelines for each institution and histological subtype are detailed in **Supplementary Table 1**. The inclusion criteria required (a) USC and EEC confirmed surgically and pathologically; (b) a pelvic MRI conducted within 14 days before gynecological surgery. The exclusion criteria encompassed: (a) maximum tumor diameter under 1 cm; (b) incomplete MRI examination; (c) incomplete pathology report; (d) presence of mixed cellular components and (e) history of neoadjuvant therapy. Ultimately, a total of 210 patients were included in the study, comprising 68 with USCs and 142 with EECs. Patients from Institution I and II were randomly assigned to a training cohort (100 patients) and an internal test cohort (44 patients) in a 7:3 ratio. A total of 66 patients were included as an external test cohort by Institutions III and IV. **Figure 1** illustrates the flowchart of the patient recruitment process.

### MRI acquisition

MRI was performed using either a 3.0-T or 1.5-T scanner with a pelvic phased-array surface coil. Institutions I and II utilized Siemens Magnetom Verio (3.0-T) and Siemens Magnetom Area (1.5-T) scanners, while institutions III and IV employed Siemens Magnetom Avanto (1.5-T) and GE Medical System Discovery HD750 (3.0-T) scanners. The sequences obtained included axial and sagittal T2-weighted imaging(T2WI), diffusion-weighted imaging (DWI) with a b-value of 800 or 1000 s/mm², and axial and sagittal contrast-enhanced MRI (CE-MRI). CE-MRI was conducted following the administration of gadolinium chelate (Gadovist, Bayer) at a dosage of 0.2 mmol/kg body weight. The detailed MRI acquisition protocols are summarized in **Supplementary Table 2**.

### Clinical and conventional MR evaluation

Clinical data were collected from medical records, encompassing age, body mass index (BMI), menopausal status, obstetric history, family history of malignancy, diabetes history, International Federation of Gynecology and Obstetrics (FIGO) stage (2023), tumor markers (CA-125, CA-199, CEA, HE4), and details of myometrial and cervical stromal invasion, adnexal involvement, parametrial invasion, lymph node metastasis, and presence of abnormal ascites. For subsequent modeling, tumor grade was categorized as follows: (a) low grade, comprising FIGO grades 1 and 2 endometrioid carcinoma, and (b) high grade, consisting of FIGO grade 3 endometrioid carcinoma or uterine serous carcinoma (8). Additionally, in accordance with European Society for Medical Oncology guidelines, FIGO stage was categorized into early (IA) and advanced (IB or higher) stages for risk stratification (28). For the purpose of baseline characterization and analysis in this study, FIGO stage and histopathologic grade were determined based on the preoperative endometrial biopsy or D&C results, reflecting the diagnostic information available at the time of initial clinical decision-making.

Two experienced radiologists, LP.L. (Reader 1) with 5 years of experience and Y.S. (Reader 2) with 8 years of experience in gynecologic imaging, independently assessed the multiparametric MR images without access to medical records or pathological data. They assessed lesion characteristics including location, borders, growth patterns, diffuse distribution, presence of necrosis and hemorrhage, tumor largest diameter, tumor volume (calculated as d1×d2×d3×π/6, where d1 and d2 are measured along and perpendicular to the uterine long axis in the sagittal plane, and d3 is the largest lateral diameter in the axial plane). Additionally, they assessed signal intensity ratios (SIR) of the tumor and gluteus maximus on T2WI, DWI, and CE-T1WI, enhancement patterns on CE-T1WI, homogeneity, and the ratios of endometrial thickness (ET) to the largest longitudinal and anteroposterior (AP) dimensions of the uterus on T2WI sagittal images (12, 29, 30)(**Supplementary Figure 1**). Features were evaluated independently by two radiologists, and any discrepancies were resolved by consensus. The inter-observer agreement for the qualitative clinical-radiological features was assessed using Cohen’s kappa (κ) statistic, and for continuous variables, the intraclass correlation coefficient (ICC) was used (**Supplementary Table 3**).

### Image segmentation and feature extraction

**Figure 2:**
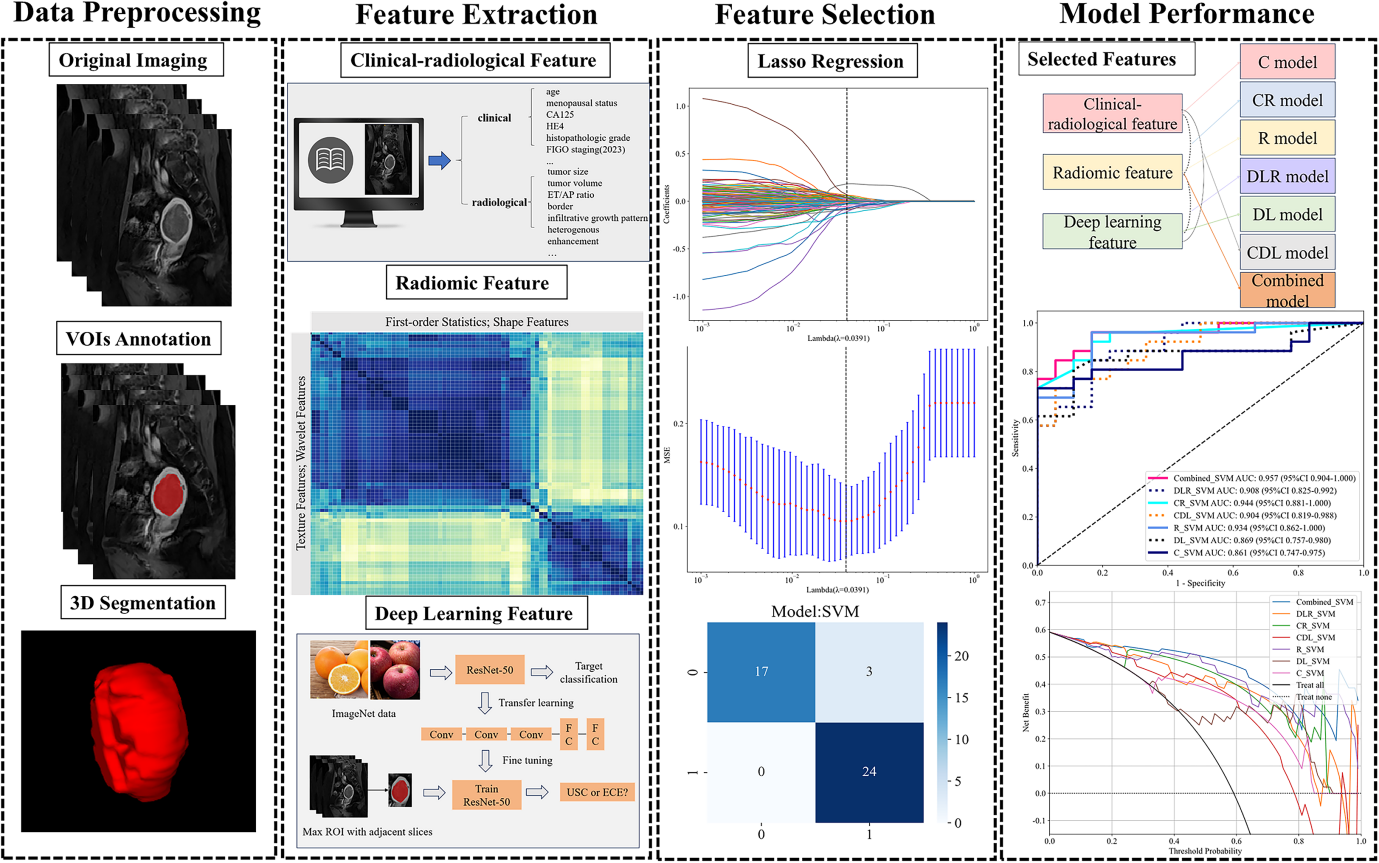
Workflow of model development. CA125, carbohydrate antigen 125; HE4, Human Epididymis Protein 4; ET/AP ratio, ratios of endometrial thickness to the largest longitudinal and anteroposterior dimensions; LASSO, least absolute shrinkage and selection operator.

**Figure 2** provides an overview of the study’s pipeline. The region of interest (ROI) was manually delineated along the lesion’s edge using ITK-SNAP software on T2WI, DWI, and CE-T1WI at the delayed phase, ensuring minimal inclusion of normal tissue to acquire comprehensive tumor data. Each tumor’s volumetric region of interest (VOI) was segmented. All ROIs drawing were performed by two experienced radiologists (Reader 1 and Reader 2) blinded to the patients’ histopathology. With 3-month intervals, 30 patients were randomly selected for Reader 2 to repeat the tumor ROI drawing. The inter-/intra-observer variability of the extracted features was assessed by ICC test. ICC > 0.75 indicated satisfactory agreement.

Radiomics analysis was conducted using PyRadiomics version 3.0.1, employing VOIs from T2WI, DWI, and delayed phase CE-T1WI. Prior to feature extraction, each image sequence was normalized by centering the gray values at the mean and scaling them according to the standard deviation, which effectively minimized variations caused by different scanners, scanning parameters, and protocols. A total of 535 radiomics features were extracted from various MRI images (T2WI, DWI, CE-T1WI), comprising 70 shape features, 90 first-order histogram features, and texture features including 120 grey level cooccurrence matrix (GLCM), 80 grey level run length matrix (GLRLM), 80 grey level size zone matrix (GLSZM), 25 neighboring grey tone difference matrix (NGTDM), and 70 grey level dependence matrix (GLDM). The study design adhered to the reporting guidelines of the Image Biomarker Standardization Initiative (IBSI) (31).

DL features were extracted utilizing a pre-trained Resnet50 convolutional neural network (CNN) model. Before extracting DL features, the data undergoes processing through these steps: (1) select the mask with the largest ROI in the labeled MRI; (2) crop MRI images using minimal bounding rectangles; (3) resize the tumor patch to 224 × 224 pixels. The Resnet50 network was initially pre-trained on the ImageNet dataset, followed by transfer learning on the training set. Upon completing Resnet50 training, we extracted 2048 deep learning features from each patch using the penultimate average pooling layer of the model. The features were then compressed to a set of 64 features using principal component analysis (PCA). Eventually, a total of 320 DL features was extracted from all series. Gradient-weighted class activation mapping (Grad-CAM) was employed to enhance model transparency and explore interpretability through visualization.

### Feature selection

We applied z-score normalization to all features and removed those with constant values. Radiomics signatures with an ICC greater than 0.75 were initially screened using the Spearman correlation test. We retained one feature for further analysis when the Spearman correlation coefficient between two features exceeded 0.9. These features were further screened using the least absolute shrinkage and selection operator (LASSO). The regularization parameter (λ) was tuned using the one-standard error of the minimum criteria (1-SE criteria) alongside tenfold cross-validation-based feature selection (see **Supplementary Figure 2**). Following feature selection, the synthetic minority oversampling technique (SMOTE) algorithm was employed on the training set, but using only the features selected by LASSO, to balance the minority class samples for the subsequent model training step.

### Model construction and validation

A SVM (support vector machine) algorithm was employed to construct seven models including a clinical-radiological model utilizing clinical and radiological data, a radiomics model using radiomics features, a DL model leveraging deep learning features, a CR model combining clinical-radiological and radiomics features, a DLR model integrating radiomics and deep learning features, a CDL model combining clinical-radiological and deep learning features, and a comprehensive all-combined model incorporating all selected features. All feature integrations were performed through direct concatenation (feature-level fusion) to maximize information utilization.

The models were developed in the training set and validated with both internal and external test sets. Model predictive performance was evaluated via a receiver operating characteristic (ROC) curve, with results presented as the area under the curve (AUC) and corresponding 95% confidence interval (CI). The accuracy (ACC), sensitivity (SEN), specificity (SPEC), and F1 score were determined using the cut-off value that maximizes the Youden index from the ROC curve analysis.

### Statistical analysis

Characteristics were compared using the independent t-test or Mann–Whitney U test for continuous variables, and Fisher’ s exact test or χ (2) test for categorical variables, with *p*-values adjusted via the Benjamini-Hochberg correction. The DeLong test was employed to compare the AUCs. Decision curve analysis (DCA) evaluated the models’ clinical utility by analyzing net benefit across various threshold probabilities in the testing sets. Statistical analyses were conducted using Python (version 3.9; https://www.python.org/), R (version 4.1.2; https://www.r-project.org/) and SPSS (version 26.0; https://www.ibm.com/). Statistical significance was defined as a two-sided *p*-value < 0.05. The Benjamini-Hochberg procedure was used to adjust for multiple testing. To assess the adequacy of the achieved sample size, a *post hoc* power analysis was conducted using G*Power software (version 3.1.9.7).

## Results

### Patient characteristics

This study enrolled 210 patients: divided into a training set of 100, an internal-testing set of 44, and an external-testing set of 66. The *post-hoc* power analysis demonstrated a statistical power of 87%, confirming that our sample size is sufficiently. A comparison between preoperative biopsy and final surgical pathology revealed discordance in 7 of 210 cases (3.3%), wherein the final diagnosis was of a higher grade or more aggressive histologic subtype than initially determined by biopsy. **Table 1** details patient characteristics within the USC and EEC groups across different cohorts. The age and proportion of postmenopausal patients were higher in the USC group compared to the EEC group (*p* < 0.05), patients with USC usually presented with higher HE4 level, FIGO staging and histopathologic grade (*p* < 0.05). Significant disparities were also observed between USC and EEC groups in terms of ET/AP ratio, tumor border, infiltrative growth pattern, diffuse distribution, presence of necrosis, inhomogeneity, heterogenous enhancement, deep myometrial invasion, cervical stromal invasion, adnexal involvement and pelvic lymph node metastasis (all *p* < 0.05).

**Table 1:** Baseline characteristics of study sets. (revised version).

Variable	Training set, N = 100			Internal-testing set, N = 44			External-testing set, N = 66			Summation, N = 210		
	EEC	USC	*p-value*^2^*	EEC	USC	*p-value*^3^*	EEC	USC	*p-value*^3^*	EEC	USC	*p-value*^2^*
	N = 72 (72%)^1^	N = 28 (28%)^1^		N = 24 (55%)^1^	N = 20 (45%)^1^		N = 44 (67%)^1^	N = 22 (33%)^1^		N = 140 (67%)^1^	N = 70 (33%)^1^	
Age	53.17 (9.51)	60.04 (12.03)	0.014	53.21 (6.69)	61.05 (9.83)	<0.05	59.27 (9.11)	66.45 (5.81)	<0.05	55.09 (9.35)	62.34 (10.08)	<0.05
BMI	24.72 [22.58, 27.30]	23.97 [21.92,25.31]	0.182	24.29 [22.72, 25.54]	25.08 [23.82, 26.84]	0.187	24.39 [23.26, 26.33]	22.61 [21.24, 26.37]	0.100	24.43 [22.74, 26.54]	24.08 [21.40, 26.31]	0.191
Menopausal status			0.057			0.045			0.039			<0.05
Premenopausal	32 (44.44%)	6 (21.43%)		10 (41.67%)	2 (10.00%)		10 (22.73%)	0 (0.00%)		52 (37.14%)	8 (11.43%)	
postmenopausal	40 (55.56%)	22 (78.57%)		14 (58.33%)	18 (90.00%)		34 (77.27%)	22 (100.00%)		88 (62.86%)	62 (88.57%)	
Reproductive History			>0.999			0.837			>0.999			0.837
Absent	4 (5.56%)	1 (3.57%)		3 (12.50%)	1 (5.00%)		1 (2.27%)	0 (0.00%)		8 (5.71%)	2 (2.86%)	
Present	68 (94.44%)	27 (96.43%)		21 (87.50%)	19 (95.00%)		43 (97.73%)	22 (100.00%)		132 (94.29%)	68 (97.14%)	
Other carcinoma			>0.999			0.837			>0.999			0.343
Absent	67 (93.06%)	27 (96.43%)		22 (91.67%)	20 (100.00%)		42 (95.45%)	21 (95.45%)		131 (93.57%)	68 (97.14%)	
Present	5 (6.94%)	1 (3.57%)		2 (8.33%)	0 (0.00%)		2 (4.55%)	1 (4.55%)		9 (6.43%)	2 (2.86%)	
Diabetes			0.723			0.738			0.143			>0.999
Absent	55 (76.39%)	23 (82.14%)		21 (87.50%)	19 (95.00%)		35 (79.55%)	13 (59.09%)		111 (79.29%)	55 (78.57%)	
Present	17 (23.61%)	5 (17.86%)		3 (12.50%)	1 (5.00%)		9 (20.45%)	9 (40.91%)		29 (20.71%)	15 (21.43%)	
Histopathologic grade			<0.05			<0.05			<0.05			<0.05
Low (grade 1 or 2)	53 (73.61%)	0 (0.00%)		15 (62.50%)	0 (0.00%)		38 (86.36%)	0 (0.00%)		106 (75.71%)	0 (0.00%)	
High (grade 3 and USCs)	19 (26.39%)	28 (100.00%)		9 (37.50%)	20 (100.00%)		6 (13.64%)	22 (100.00%)		34 (24.29%)	70 (100.00%)	
FIGO staging (2023)			<0.05			<0.05			<0.05			<0.05
Ia	44 (61.11%)	0 (0.00%)		16 (66.67%)	0 (0.00%)		30 (68.18%)	0 (0.00%)		90 (64.29%)	0 (0.00%)	
Ib or higher	28 (38.89%)	28 (100.00%)		8 (33.33%)	20 (100.00%)		14 (31.82%)	22 (100.00%)		50 (35.71%)	70 (100.00%)	
CA125	23.50 [14.75, 37.65]	18.80 [15.14,39.52]	0.893	17.30 [12.38, 29.33]	18.38 [13.90, 25.06]	0.906	20.45 [11.61, 28.61]	53.99 [17.23, 130.68]	0.005	21.60 [13.70, 32.93]	20.61 [14.85, 54.21]	0.167
CA199	14.80 [8.38, 30.03]	14.79 [10.24,20.49]	0.721	12.55 [7.43, 18.30]	14.08 [6.85, 23.09]	0.588	19.76 [12.61, 39.07]	20.53 [12.89, 32.14]	0.698	15.40 [9.18, 30.03]	15.75 [9.83, 24.40]	0.734
CEA	1.55 [1.18, 2.23]	1.85 [1.31, 2.40]	0.365	1.45 [1.18, 2.13]	1.67 [1.10, 2.33]	0.953	1.50 [1.18, 2.13]	1.74 [1.28, 2.90]	0.139	1.50 [1.18, 2.20]	1.75 [1.20, 2.70]	0.176
HE4	78.06 [56.33,107.4]	99.90 [69.75,146.3]	0.24	77.04 [62.98,122.25]	77.50 [60.16,100.30]	0.939	79.38 [61.49,127.31]	178.20 [126.10, 286.15]	<0.05	77.90 [59.18, 116.36]	107.40 [70.85, 197.98]	<0.05
Tumor size	3.32 [2.53, 4.86]	3.38 [2.55, 4.58]	0.939	3.13 [1.93, 4.60]	3.19 [2.14, 4.38]	0.939	3.21 [2.02, 4.31]	3.96 [3.13, 5.46]	0.240	3.29 [2.22, 4.75]	3.51 [2.47, 4.92]	0.837
Tumor Volume	7.83 [2.28, 18.19]	9.06 [4.53, 26.26]	0.501	9.02 [1.71, 26.15]	6.86 [2.64, 15.38]	0.972	6.24 [1.17, 15.93]	13.86 [4.78, 35.38]	0.048	7.36 [1.95, 18.19]	10.82 [3.71, 26.43]	0.121
ET/AP ratio	0.36 [0.27, 0.44]	0.52 [0.36, 0.70]	<0.05	0.35 [0.26, 0.49]	0.53 [0.33, 0.67]	0.048	0.46 [0.33, 0.59]	0.66 [0.52, 0.79]	0.044	0.38 [0.28, 0.51]	0.56 [0.36, 0.70]	<0.05
SIR-CE-T1WI	1.36 [1.22, 1.55]	1.69 [1.45, 1.95]	0.006	1.43 [1.33, 1.66]	1.66 [1.41, 1.98]	0.244	1.39 [1.20, 1.58]	0.97 [0.64, 1.63]	0.062	1.40 [1.23, 1.60]	1.59 [1.10, 1.95]	0.121
SIR-T2WI	2.06 [1.59, 2.36]	1.75 [1.29, 2.13]	0.188	1.94 [1.66, 2.26]	1.85 [1.52, 3.01]	0.888	2.10 [1.74, 2.55]	1.65 [1.32, 2.42]	0.033	2.04 [1.65, 2.42]	1.77 [1.35, 2.44]	0.106
SIR-DWI	5.07 [4.21, 6.20]	6.08 [4.28, 7.37]	0.274	4.98 [4.16, 7.39]	5.07 [3.86, 6.48]	0.502	3.95 [3.09, 4.44]	3.67 [2.86, 4.71]	0.984	4.67 [3.74, 5.63]	4.74 [3.57, 7.19]	0.651
location			0.007			0.347			0.347			0.143
Cornua uteri	6 (8.33%)	1 (3.57%)		4 (16.67%)	2 (10.00%)		5 (11.36%)	1 (4.55%)		15 (10.71%)	4 (5.71%)	
Fundus of uterus	18 (25.00%)	0 (0.00%)		6 (25.00%)	2 (10.00%)		6 (13.64%)	6 (27.27%)		30 (21.43%)	8 (11.43%)	
Corpus uteri	48 (66.67%)	27 (96.43%)		14 (58.33%)	16 (80.00%)		33 (75.00%)	15 (68.18%)		95 (67.86%)	58 (82.86%)	
Border			0.007			0.079			0.305			<0.05
Well-defined	38 (52.78%)	4 (14.29%)		12 (50.00%)	3 (15.00%)		16 (36.36%)	4 (18.18%)		66 (47.14%)	11 (15.71%)	
Ill-defined	34 (47.22%)	24 (85.71%)		12 (50.00%)	17 (85.00%)		28 (63.64%)	18 (81.82%)		74 (52.86%)	59 (84.29%)	
Infiltrative Growth pattern			0.037			0.367			0.198			<0.05
Absent	28 (38.89%)	3 (10.71%)		8 (33.33%)	3 (15.00%)		13 (29.55%)	2 (9.09%)		49 (35.00%)	8 (11.43%)	
Present	44 (61.11%)	25 (89.29%)		16 (66.67%)	17 (85.00%)		31 (70.45%)	20 (90.91%)		91 (65.00%)	62 (88.57%)	
Diffuse distribution			<0.05			0.010			0.929			<0.05
Absent	49 (68.06%)	7 (25.00%)		17 (70.83%)	4 (20.00%)		18 (40.91%)	8 (36.36%)		84 (60.00%)	19 (27.14%)	
Present	23 (31.94%)	21 (75.00%)		7 (29.17%)	16 (80.00%)		26 (59.09%)	14 (63.64%)		56 (40.00%)	51 (72.86%)	
Presence of necrosis			0.367			0.771			0.108			0.037
Absent	58 (80.56%)	19 (67.86%)		18 (75.00%)	13 (65.00%)		37 (84.09%)	13 (59.09%)		113 (80.71%)	45 (64.29%)	
Present	14 (19.44%)	9 (32.14%)		6 (25.00%)	7 (35.00%)		7 (15.91%)	9 (40.91%)		27 (19.29%)	25 (35.71%)	
Presence of hemorrhage			>0.999			>0.999			0.005			0.11
Absent	62 (86.11%)	24 (85.71%)		19 (79.17%)	15 (75.00%)		44 (100.00%)	16 (72.73%)		125 (89.29%)	55 (78.57%)	
Present	10 (13.89%)	4 (14.29%)		5 (20.83%)	5 (25.00%)		0 (0.00%)	6 (27.27%)		15 (10.71%)	15 (21.43%)	
Inhomogeneity on T2WI			0.013			0.435			0.421			0.005
Absent	50 (69.44%)	10 (35.71%)		15 (62.50%)	9 (45.00%)		23 (52.27%)	8 (36.36%)		88 (62.86%)	27 (38.57%)	
Present	22 (30.56%)	18 (64.29%)		9 (37.50%)	11 (55.00%)		21 (47.73%)	14 (63.64%)		52 (37.14%)	43 (61.43%)	
Heterogenous enhancement			0.171			0.563			0.110			0.018
Absent	45 (62.50%)	12 (42.86%)		14 (58.33%)	9 (45.00%)		24 (54.55%)	6 (27.27%)		83 (59.29%)	27 (38.57%)	
Present	27 (37.50%)	16 (57.14%)		10 (41.67%)	11 (55.00%)		20 (45.45%)	16 (72.73%)		57 (40.71%)	43 (61.43%)	
Myometrial Invasion			0.179			0.301			0.179			0.032
<50%	47 (65.28%)	13 (46.43%)		17 (70.83%)	10 (50.00%)		30 (68.18%)	10 (45.45%)		94 (67.14%)	33 (47.14%)	
≥47.	25 (34.72%)	15 (53.57%)		7 (29.17%)	10 (50.00%)		14 (31.82%)	12 (54.55%)		46 (32.86%)	37 (52.86%)	
Cervical Stromal invasion			0.170			0.495			0.170			0.024
Absent	67 (93.06%)	22 (78.57%)		22 (91.67%)	16 (80.00%)		43 (97.73%)	18 (81.82%)		132 (94.29%)	56 (80.00%)	
Present	5 (6.94%)	6 (21.43%)		2 (8.33%)	4 (20.00%)		1 (2.27%)	4 (18.18%)		8 (5.71%)	14 (20.00%)	
Parametrial extension			>0.999			0.556			0.457			0.662
Absent	70 (97.22%)	28 (100.00%)		24 (100.00%)	19 (95.00%)		44 (100.00%)	21 (95.45%)		138 (98.57%)	68 (97.14%)	
Present	2 (2.78%)	0(0.00%)		0 (0.00%)	1 (5.00%)		0 (0.00%)	1 (4.55%)		2 (1.43%)	2 (2.86%)	
Adnexal involvement			0.016			0.432			0.039			<0.05
Absent	69 (95.83%)	21 (75.00%)		22 (91.67%)	15 (75.00%)		44 (100.00%)	18 (81.82%)		135 (96.43%)	54 (77.14%)	
Present	3 (4.17%)	7 (25.00%)		2 (8.33%)	5 (25.00%)		0 (0.00%)	4 (18.18%)		5 (3.57%)	16 (22.86%)	
Pelvic lymph node metastasis			0.040			0.974			<0.05			<0.05
Absent	66 (91.67%)	20 (71.43%)		18 (75.00%)	16 (80.00%)		44 (100.00%)	13 (59.09%)		128 (91.43%)	49 (70.00%)	
Present	6 (8.33%)	8 (28.57%)		6 (25.00%)	4 (20.00%)		0 (0.00%)	9 (40.91%)		12 (8.57%)	21 (30.00%)	
Abnormal ascites			>0.999			0.387			>0.999			0.387
Absent	71 (98.61%)	28 (100.00%)		24 (100.00%)	18 (90.00%)		44 (100.00%)	22 (100.00%)		139 (99.29%)	68 (97.14%)	
Present	1 (1.39%)	0 (0.00%)		0 (0.00%)	2 (10.00%)		0 (0.00%)	0 (0.00%)		1 (0.71%)	2 (2.86%)	
Peritoneal dissemination			0.228			0.455			0.400			0.072
Absent	72 (100.00%)	26 (92.86%)		24 (100.00%)	19 (95.00%)		44 (100.00%)	21 (95.45%)		140 (100.00%)	66 (94.29%)	
Present	0 (0.00%)	2(7.14%)		0 (0.00%)	1 (5.00%)		0 (0.00%)	1 (4.55%)		0 (0.00%)	4 (5.71%)	

^1^Mean (SD); Median [IQR]; n (%).

^2^Wilcoxon rank sum test; Pearson’s Chi-squared test; Fisher’s exact test

^3^Wilcoxon rank sum test; Wilcoxon rank sum exact test; Pearson’s Chi-squared test; Fisher’s exact test

*The Benjamini-Hochberg procedure was used to adjust for multiple testing.

### Development and validation of clinical-radiological, radiomics, DL and combined models

Among the 17 clinical-radiological characteristics, histopathologic grade, FIGO staging, ET/AP ratio and diffuse distribution were identified as significant features using the LASSO algorithm (**Supplementary Figure 3A**). The mean inter- and intra-observer reliabilities were 0.821 (95% CI 0.726–0.896) and 0.859 (95% CI 0.773–0.912), indicating excellent consistency in radiomics features. A total of 194 radiomics features and 160 DL features of the tumor, each with Spearman correlation coefficients > 0.9, were retained for further selection. Using LASSO algorithms, 30 radiomics features and 14 DL features were selected to construct the radiomics, DL, and combined models. **Supplementary Figure 3** provides additional information on the features chosen by the LASSO algorithm.

**Figure 3:**
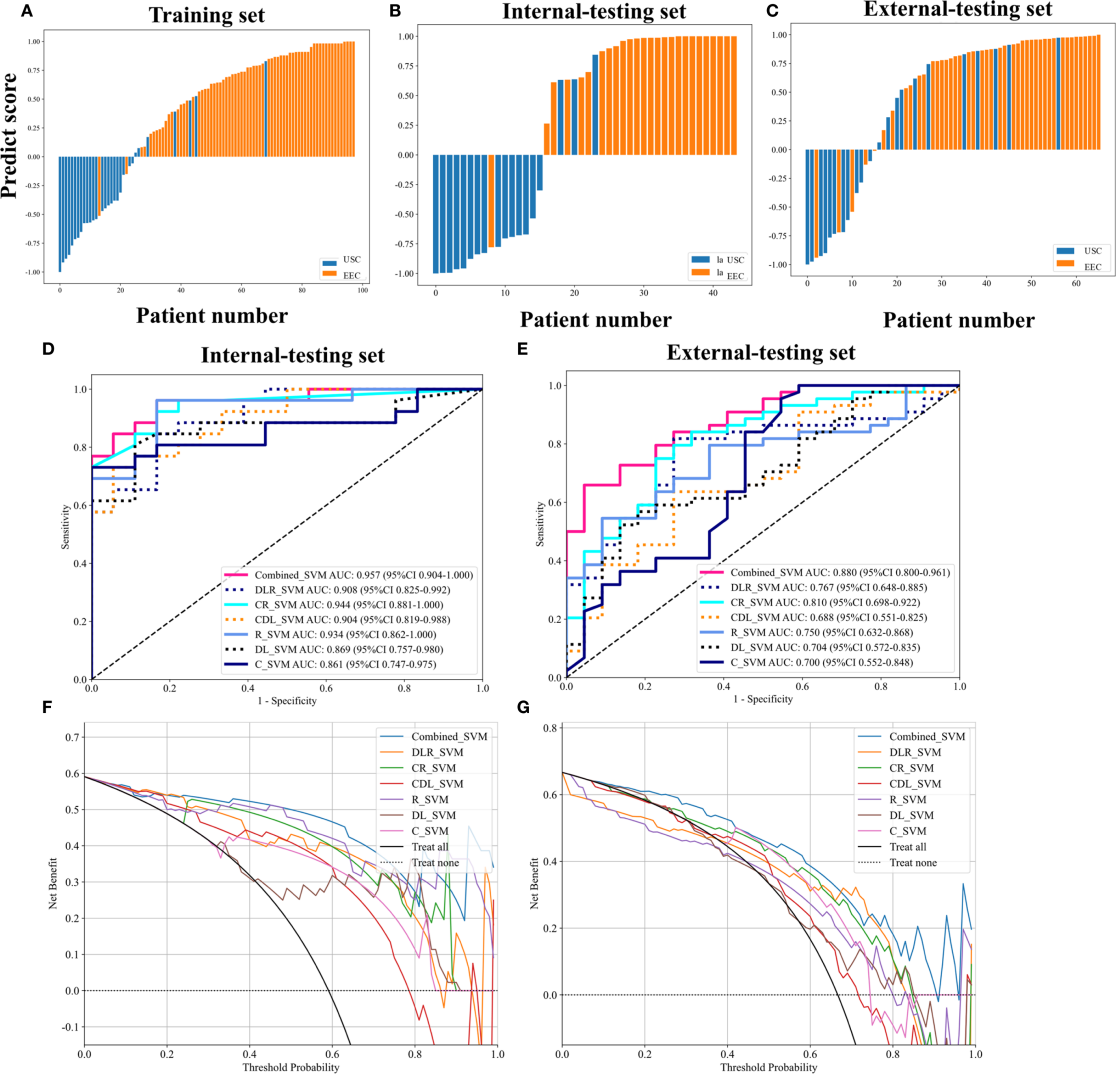
Patient predict scores output by the combined model in the training and testing sets **(A–C)**. Receiver operation characteristic (ROC) curves of different models in the internal-testing set and external-testing set **(D, E)**. The all-combined model had the best discriminating ability among seven models, with an area under the curve (AUC) of 0.957 in the internal-testing set and 0.880 in the external-testing set. Decision curve analysis (DCA) of the different models in the internal-testing set and external-testing set **(F, G)**. The x-axis means the high-risk threshold, and the y-axis means clinic net benefit.

The SVM model was optimized using the training set and subsequently evaluated on both internal and external test sets. **Figures 3A–C** displays the predicted scores for patients, demonstrating the models’ strong classification capability. **Table 2** presents the performance metrics of various models on both the training and testing datasets. The clinical-radiological model achieved AUCs of 0.861 (95% CI: 0.747-0.975) and 0.700 (95% CI: 0.552-0.848) in the internal and external testing set, respectively. The AUCs of the radiomics model were 0.934 (95% CI: 0.862-0.999) and 0.750 (95% CI: 0.632-0.868) in the internal and external testing set, respectively. The AUCs of the DL model were 0.869 (95% CI: 0.757-0.980) in the internal-testing set, and 0.704 (95% CI:0.572-0.835) in the external-testing set. The all-combined model showed excellent predictive performance. The all-combined model demonstrated superior classification performance in the internal-testing set with an AUC of 0.957 (95% CI: 0.904-1.000), accuracy of 0.886, sensitivity of 0.923, specificity of 0.833, and F1 score of 0.906, while in the external-testing set, these values were 0.880 (95% CI: 0.800-0.961), 0.742, 0.636, 0.955, and 0.767, respectively.

**Table 2:** Performances of the predictive models in the training and test sets. (revised version).

Model name		AUC (95%CI)	Accuracy (95%CI)	Sensitivity (95%CI)	Specificity (95%CI)	F1 score (95%CI)
Clinical-radiological	Training	0.910 (0.852-0.968)	0.85 (0.772-0.928)	0.886 (0.815-0.957)	0.767 (0.657-0.877)	0.892 (0.828-0.956)
	Internal-test	0.861 (0.747-0.975)	0.818 (0.704-0.932)	0.692 (0.519-0.865)	0.999 (0.993-1.000)	0.818 (0.704-0.932)
	External-test	0.700 (0.552-0.848)	0.773 (0.669-0.877)	0.555 (0.401-0.709)	0.647 (0.492-0.802)	0.845 (0.756-0.934)
Radiomics	Training	0.977 (0.955-0.999)	0.920 (0.865-0.975)	0.903 (0.836-0.970)	0.964 (0.917-1.000)	0.942 (0.897-0.987)
	Internal-test	0.934 (0.862-0.999)	0.886 (0.800-0.972)	0.833 (0.698-0.968)	0.889 (0.784-0.994)	0.906 (0.826-0.986)
	External-test	0.750 (0.632-0.868)	0.652 (0.534-0.770)	0.523 (0.372-0.674)	0.909 (0.818-1.000)	0.667 (0.543-0.791)
DL	Training	0.976 (0.950-0.999)	0.930 (0.882-0.978)	0.900 (0.831-0.969)	1.000 (1.000-1.000)	0.947 (0.905-0.989)
	Internal-test	0.869 (0.757-0.980)	0.818 (0.704-0.932)	0.769 (0.614-0.924)	0.889 (0.784-0.994)	0.833 (0.723-0.943)
	External-test	0.704 (0.572-0.835)	0.636 (0.518-0.754)	0.545 (0.394-0.696)	0.818 (0.691-0.945)	0.667 (0.543-0.791)
Clinical-radiological + radiomics	Training	0.984 (0.950-0.999)	0.910 (0.852-0.968)	0.889 (0.821-0.957)	0.964 (0.917-1.000)	0.934 (0.885-0.983)
	Internal-test	0.944 (0.881-0.999)	0.841 (0.731-0.951)	0.846 (0.715-0.977)	0.833 (0.698-0.968)	0.863 (0.769-0.957)
	External-test	0.810 (0.698-0.922)	0.742 (0.638-0.846)	0.727 (0.589-0.865)	0.773 (0.639-0.907)	0.790 (0.683-0.897)
Clinical-radiological + DL	Training	0.918 (0.858-0.978)	0.860 (0.789-0.931)	0.833 (0.747-0.919)	0.929 (0.863-0.995)	0.896 (0.833-0.959)
	Internal-test	0.904 (0.819-0.988)	0.795 (0.673-0.917)	0.692 (0.519-0.865)	0.944 (0.857-1.000)	0.800 (0.674-0.926)
	External-test	0.688 (0.551-0.825)	0.652 (0.534-0.770)	0.614 (0.466-0.762)	0.727 (0.591-0.863)	0.701 (0.579-0.823)
DL + radiomics	Training	0.999 (0.995-1.000)	0.980 (0.957-1.000)	0.972 (0.937-1.000)	1.000 (1.000-1.000)	0.986 (0.970-1.000)
	Internal-test	0.908 (0.824-0.991)	0.818 (0.704-0.932)	0.808 (0.667-0.949)	0.833 (0.698-0.968)	0.840 (0.733-0.947)
	External-test	0.767 (0.648-0.885)	0.773 (0.669-0.877)	0.795 (0.667-0.923)	0.727 (0.591-0.863)	0.824 (0.725-0.923)
Clinical-radiological + radiomics + DL	Training	0.994 (0.984-1.000)	0.970 (0.937-1.000)	0.986 (0.963-1.000)	0.929 (0.863-0.995)	0.979 (0.959-0.999)
	Internal-test	0.957 (0.904-1.000)	0.886 (0.800-0.972)	0.923 (0.829-1.000)	0.833 (0.698-0.968)	0.906 (0.826-0.986)
	External-test	0.880 (0.800-0.961)	0.742 (0.638-0.846)	0.636 (0.486-0.786)	0.955 (0.887-1.000)	0.767 (0.659-0.875)

### Comparison of the clinical-radiological, radiomics, DL and combined models

DeLong’s test indicated that the all-combined model demonstrated significantly superior discriminatory ability compared to both the clinical-radiological model (AUC = 0.880 vs. 0.700, *p* < 0.05) and DL model (AUC = 0.880 vs. 0.704, *p* < 0.05) in the external-testing set (**Figure 3E**; **Supplementary Figure 4**).

**Figure 4:**
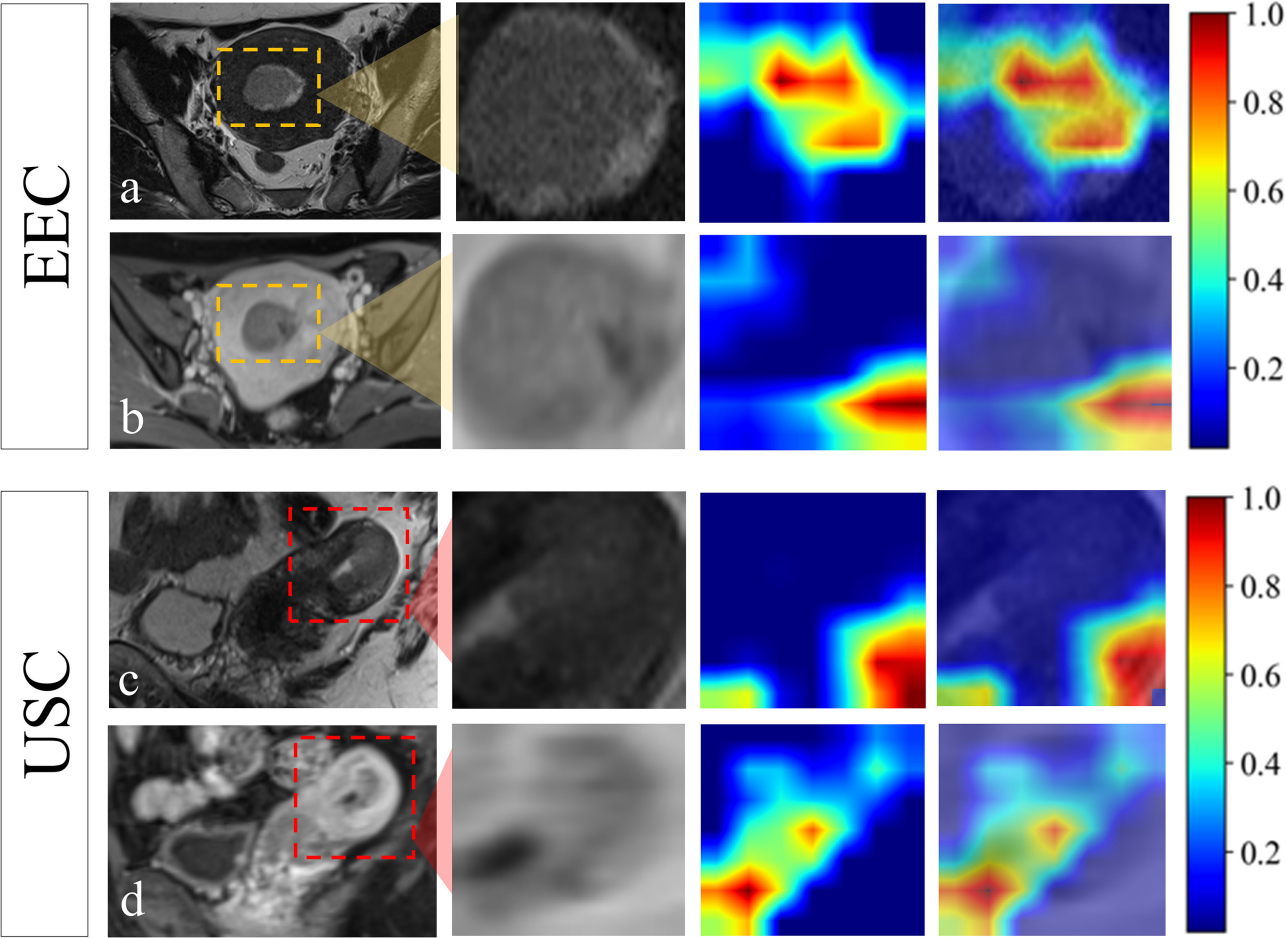
Visualization of the attention regions by the deep convolutional neural network of a 55-year-old patient who was confirmed EEC **(A, B)** and a 67-year-old patient who was confirmed USC **(C, D)**. The red and yellow regions represent the areas with higher activation, whereas the blue and green regions represent the areas with lower activation.

The all-combined model demonstrated significantly superior discriminatory power compared to the CR model (AUC = 0.880 vs. 0.810, *p* < 0.05) and CDL model in the external-testing set (AUC = 0.880 vs. 0.688, *p* < 0.05) (refer to **Table 2**; **Supplementary Figure 4**). The DLR model demonstrated superior predictive performance compared to the clinical-radiological model, although the differences were not statistically significant in both the internal-testing set (AUC = 0.908 vs. 0.861, *p* = 0.504), and the external-testing set (AUC = 0.767 vs. 0.700, *p* = 0.499) (**Figures 3D, E**; **Supplementary Figure 4**). Accuracy, sensitivity and specificity values varied across models, with the best performance in combined models such as DLR model (accuracy of 0.980, sensitivity of 0.972 and specificity of 1.000 in training) and all-combined model (accuracy of 0.742, sensitivity of 0.923 and specificity of 0.833 in the external test set). These models consistently outperformed individual models like R model (sensitivity of 0.652 in the external test set) and C model (specificity of 0.647 in the external test set). The all-combined model and DLR achieved the highest F1 scores, with the all-combined model attaining 0.979 during training and 0.906 in the internal test set. The decision curves (**Figures 3F, G**) demonstrated that the combined model provided a superior overall net benefit across most reasonable threshold probabilities in both the internal and external testing sets. **Figure 4** illustrates the activation maps highlighting image regions that significantly contribute to the feature output recognized by the deep CNN. Overall, the use of a multiparametric model based on radiomics and DL had better predictive value in the preoperative differential diagnosis between USC and EEC.

## Discussion

In contrast to EEC, USC is characterized by a high propensity for metastasis and recurrence, even in its early stages (6). Thus, the accurate and noninvasive classification of USC and EEC is vital in clinical practice. Our retrospective multicenter study revealed that combining the radiomics and DL features extracted from multiparametric MRI with clinical-radiological features could enhance the preoperative differential diagnosis accuracy between USC and EEC.

In this study, we observed that USC was more prevalent in postmenopausal women and associated with elevated HE4 levels, advanced FIGO staging, and higher histopathological grades. These findings underscore the aggressive nature of USC and align with results from other studies (7, 32, 33). Previous studies have reported notably higher median levels of CA125 and HE4 in endometrial cancer patients compared to healthy controls (34, 35). Our study found that serum HE4 levels were significantly higher in USC patients compared to EEC patients (*p* < 0.001) while no significant difference was observed in CA125 levels. This indicates that HE4 could be a more effective tumor marker for differential diagnosis in EC, complementing existing diagnostic approaches that combine ultrasonographic and inflammatory markers (34, 36, 37). Additionally, elevated serum HE4 levels may correlate with age, deeper myometrial invasion, extrauterine disease, and poorer prognosis (34, 36, 38), reinforcing its clinical utility in risk stratification. To date, only one research has primarily focused on conventional MRI signs to differentiate between USC and EEC (11), with findings indicating that USC often presents a heterogeneous signal, peritoneal dissemination, and abnormal ascites, aligning with our observations. Expanding upon these findings, our study identified the imaging characteristics of USC as exhibiting aggressive biological behaviors, including a higher ET/AP ratio, ill-defined tumor borders, infiltrative growth patterns, diffuse distribution, deep myometrial invasion, cervical stromal invasion, adnexal involvement, pelvic lymph node metastasis, and peritoneal dissemination. Additionally, USC displayed heterogeneous imaging features characterized by necrosis, inhomogeneity, and heterogeneous enhancement. By integrating histopathologic grade, FIGO staging, ET/AP ratio, and diffuse distribution identified through the LASSO algorithm, our clinical-radiological model demonstrated strong diagnostic performance in differentiating USC from EEC, with an AUC of 0.861 in the internal test set and 0.700 in the external test set. This multimodal approach echoes the emerging trend in endometrial cancer diagnostics that combines imaging parameters with laboratory biomarkers to improve diagnostic accuracy (37–39).

In our study, we utilized whole-volume multiparametric MRI radiomics features extracted from multicenter data to enhance diagnostic accuracy and provide comprehensive insights into tumor heterogeneity (17, 18). The radiomics model, which included 15 features from CE-T1WI, 10 from T2WI images, and 5 from DWI, demonstrated moderate performance, achieving AUC values of 0.934 and 0.750 in the internal and external testing sets, respectively. The high number of features derived from CE-T1WI underscores its advantages over other imaging modalities, as it offers better tissue differentiation and contrast resolution, allowing for more precise characterization of the tumor’s morphological and vascular features. This results in a greater ability to capture relevant radiomic features indicative of tumor biology and behavior. Moreover, our findings suggest that the T2WI sequence may play a crucial role in non-enhanced MRI protocols for diagnosing endometrial diseases, providing excellent contrast and spatial resolution that facilitate detailed visualization of anatomical features which is crucial for accurate diagnosis and evaluation, consistent with previous reports (39, 40). Additionally, the largest subset of features in our radiomics model was extracted from the gray-level co-occurrence matrix (GLCM) and related analyses, providing critical insights into the histopathological characteristics of endometrial cancer, facilitating the differentiation of tumor grades and aggressiveness. By evaluating features such as inverse variance, cluster shade, and zone percentage, clinicians can better understand the tumor’s structural complexity and its potential impact on prognosis and treatment decisions.

Recent advances in DL have demonstrated its considerable potential in gynecologic oncologic imaging, with studies showing its ability to detect intricate patterns in medical images and achieve diagnostic accuracy comparable to or even surpassing human experts (22, 41–43). In our study, both radiomics and DL features were extracted from the same manually segmented volumes of interest. However, they represent fundamentally different paradigms of image analysis. Handcrafted radiomics relies on pre-defined mathematical descriptors (e.g., texture, shape, first-order statistics) to quantify explicit tumor characteristics, offering high interpretability. In contrast, the deep learning approach processes raw image data through multiple convolutional and nonlinear layers, autonomously learning hierarchical, spatially contextual, and often abstract features that are not captured by conventional radiomics frameworks (18). The model integrating both feature types (DLR) demonstrated superior performance compared to models using either alone on the external-testing set (AUC = 0.767 vs. 0.750 for radiomics and 0.704 for DL), suggesting their features are complementary. This complementarity was further supported by the observation that the radiomics model achieved higher specificity (0.909 vs. 0.818) while the DL model showed higher sensitivity (0.545 vs. 0.523) in the external-testing set. We posit that while radiomics effectively quantifies known morphological patterns, DL may capture more subtle and complex spatial hierarchies within the tumor, contributing unique discriminatory information for differentiating USC from EEC. Notably, in our cohort, the model based solely on traditional radiomics features outperformed the DL model. This observation contrasts with some previous studies that have reported the superiority of DL over radiomics (30, 44, 45). We hypothesize that this discrepancy may be attributed to the data-hungry nature of deep learning; convolutional neural networks typically require large-scale datasets to effectively learn complex and robust spatial features (46). Our limited sample size, particularly for the minority USC class, may have constrained the DL model’s performance and increased its susceptibility to overfitting (47). This finding underscores the importance of dataset size and characteristics when selecting and developing AI methodologies for medical imaging tasks.

The proposed all-combined model exhibited superior performance, with an AUC of 0.957 in the internal-testing set and 0.880 in the external-testing set. It effectively characterizes intratumoral heterogeneity from medical images across various levels in a noninvasive and robust manner, thereby providing valuable insights into cancer (45, 48, 49). The integration of high-dimensional features enhances sensitivity in disease diagnosis and prediction, offering detailed information for clinicians (20). The sensitivity of our model necessitates that it be applied as a decision-support tool within a multidisciplinary framework. A negative output should not preclude comprehensive staging surgery when clinical suspicion, biopsy results, or conventional imaging features suggest an aggressive tumor. Its primary value lies in its high specificity, which can provide robust supporting evidence for managing cases with ambiguous preoperative findings. To the best of our knowledge, this study is the first to apply the DL features and traditional radiomic features for differentiating USC from EEC. Our study is distinguished by utilizing the largest sample size to date and employing an independent external-testing set for model validation, achieving satisfactory prediction efficiency. By providing clinicians with a reliable tool for personalized treatment stratification, our model complements existing AI systems for endometrial cancer detection and risk assessment (43, 50), ultimately contributing to a more comprehensive AI-powered diagnostic ecosystem for endometrial cancer management.

Our study has several limitations. First, its retrospective design carries an inherent risk of selection bias, as only patients undergoing surgical resection were included, thereby excluding those with inoperable advanced disease or conservative management—potentially limiting generalizability. Second, despite protocol harmonization, inter-scanner variability across institutions may introduce information bias and residual batch effects, which although mitigated through normalization and feature stability analysis, remains a concern. Third, the manual ROI delineation is inherently subjective; we minimized inter-observer variability by using only features with high agreement (ICC > 0.75), but fully automated segmentation is needed in the future. Fourth, while we adjusted for key confounders in our model, residual confounding from unmeasured factors remains possible. Fifth, additional sensitivity analyses, such as employing alternative feature selection methods or machine learning algorithms, could further reinforce robustness. Finally, the potential for overfitting remains a limitation due to the high dimensionality of radiomics and deep learning features relative to our sample size, particularly for the rare USC subtype. Further prospective validation in larger, multi-centric cohorts is essential to confirm the ultimate generalizability of our model.

## Conclusion

In conclusion, based on our dataset, this study demonstrates that this predictive model, which integrates multiparametric-MRI radiomics, deep learning features and clinical-radiological features, can effectively distinguish between USCs and EECs. The findings from this study could significantly inform clinical decision-making, ultimately leading to more personalized treatment strategies and improved patient outcomes for EC.

## Data Availability

The raw data supporting the conclusions of this article will be made available by the authors, without undue reservation.
