# Supplementary material for: Multiparametric MRI-based radiomics and deep learning for differentiating uterine serous carcinoma from endometrioid carcinoma: a multicenter retrospective study
Source: Front Oncol. 2025 Oct 8;15:1655384. doi: 10.3389/fonc.2025.1655384 (PMC12540183; doi:10.3389/fonc.2025.1655384)
Supplement: Supplementary file 1 [file DataSheet1.docx]

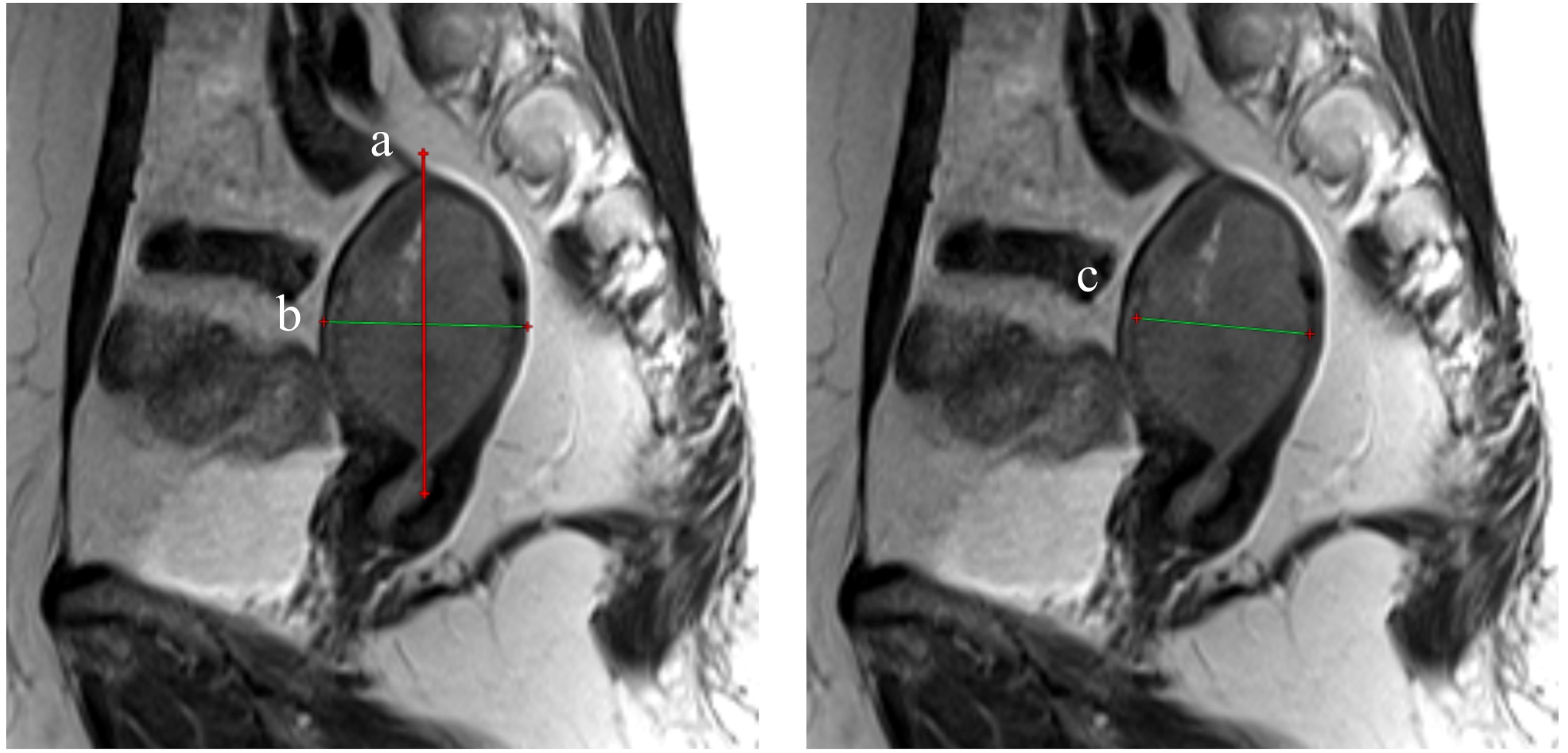


Supplementary Fig.1 Sagittal midline image of a patient with endometrioid carcinoma. a, The largest longitudinal axis of the uterus; b, maximum AP diameter of the uterus (42 mm). (b) C, maximum ET (35 mm). The calculated ET: AP is 35/42 = 0.83.


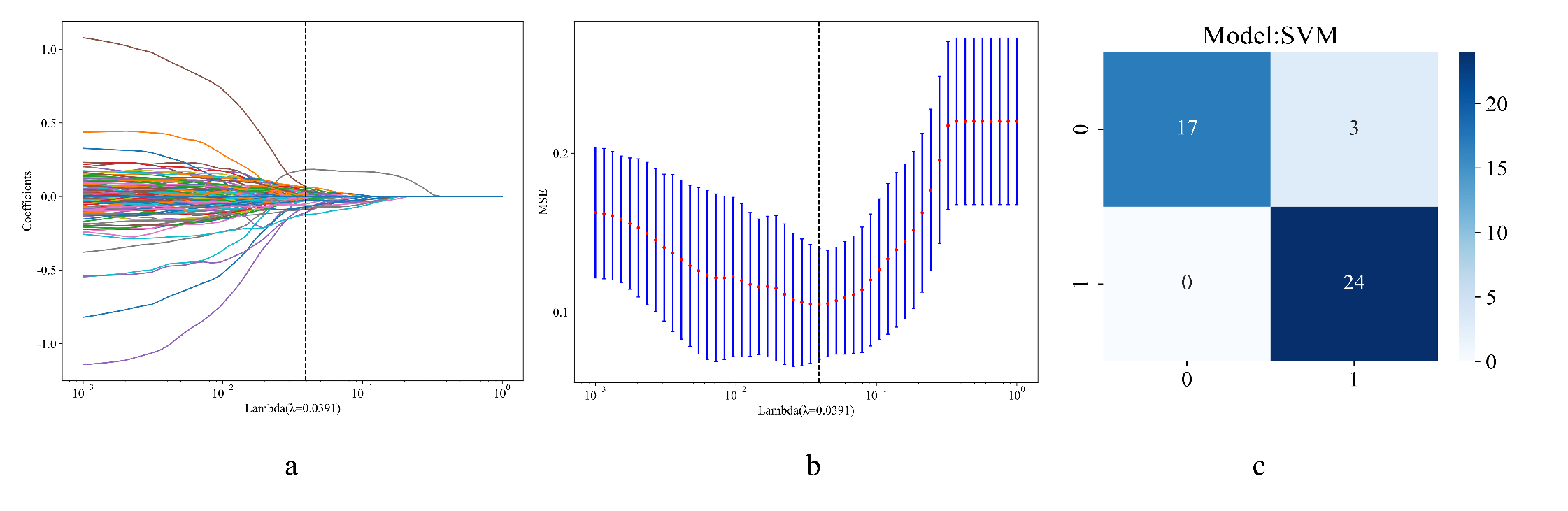


Supplementary Fig.2 The least absolute shrinkage and selection operator (LASSO) algorithm was applied to avoid overfitting (a). The 1-standard error of the minimum criteria (the 1-SE criteria) was used to tune the regularization parameter (b). The confusion matrix summarizes the model's performance on the internal-testing set (c).


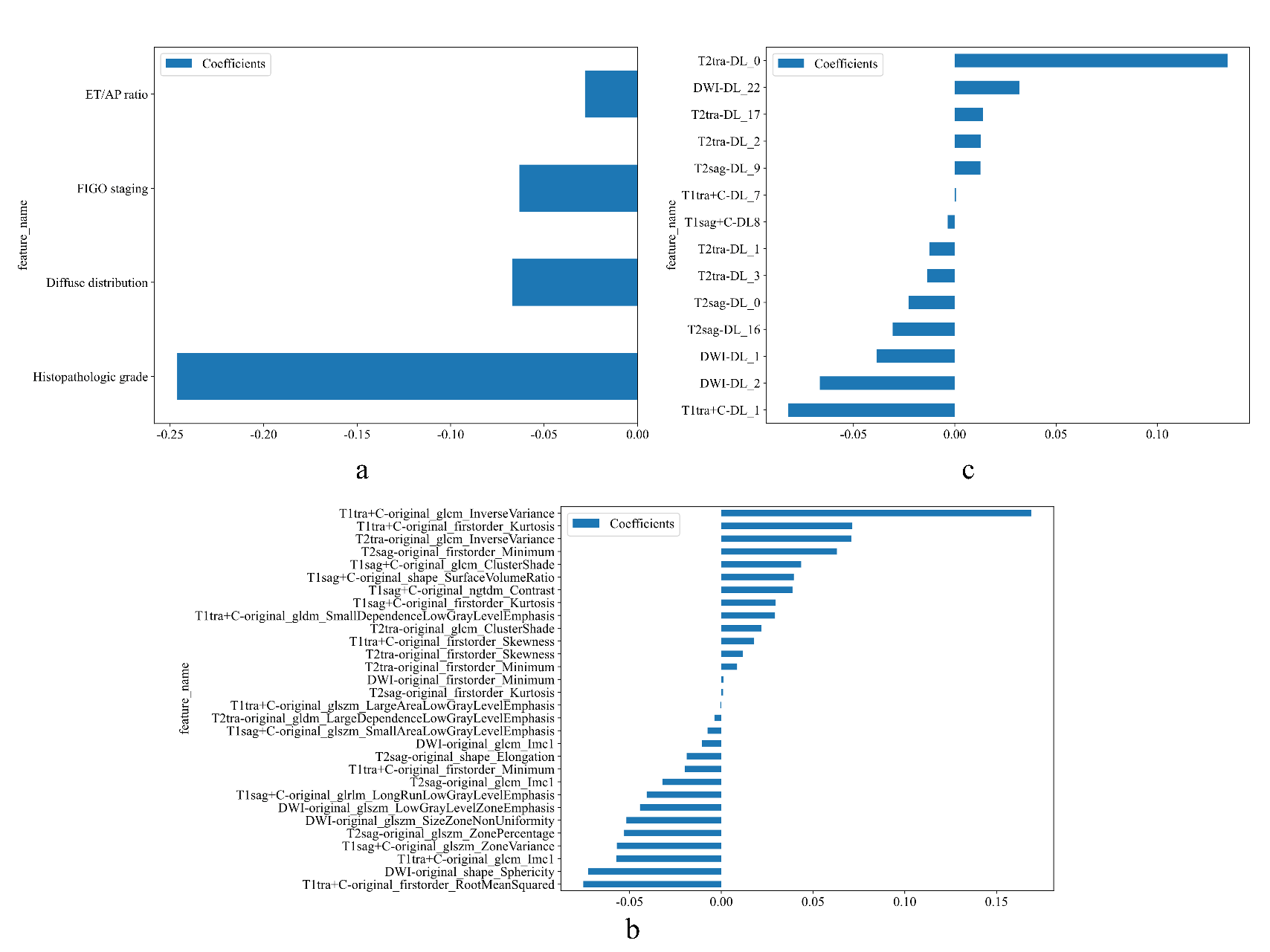


Supplementary Fig.3 Key features of clinical-radiological model (a), radiomics model (b) and deep learning model (c) after LASSO regression.


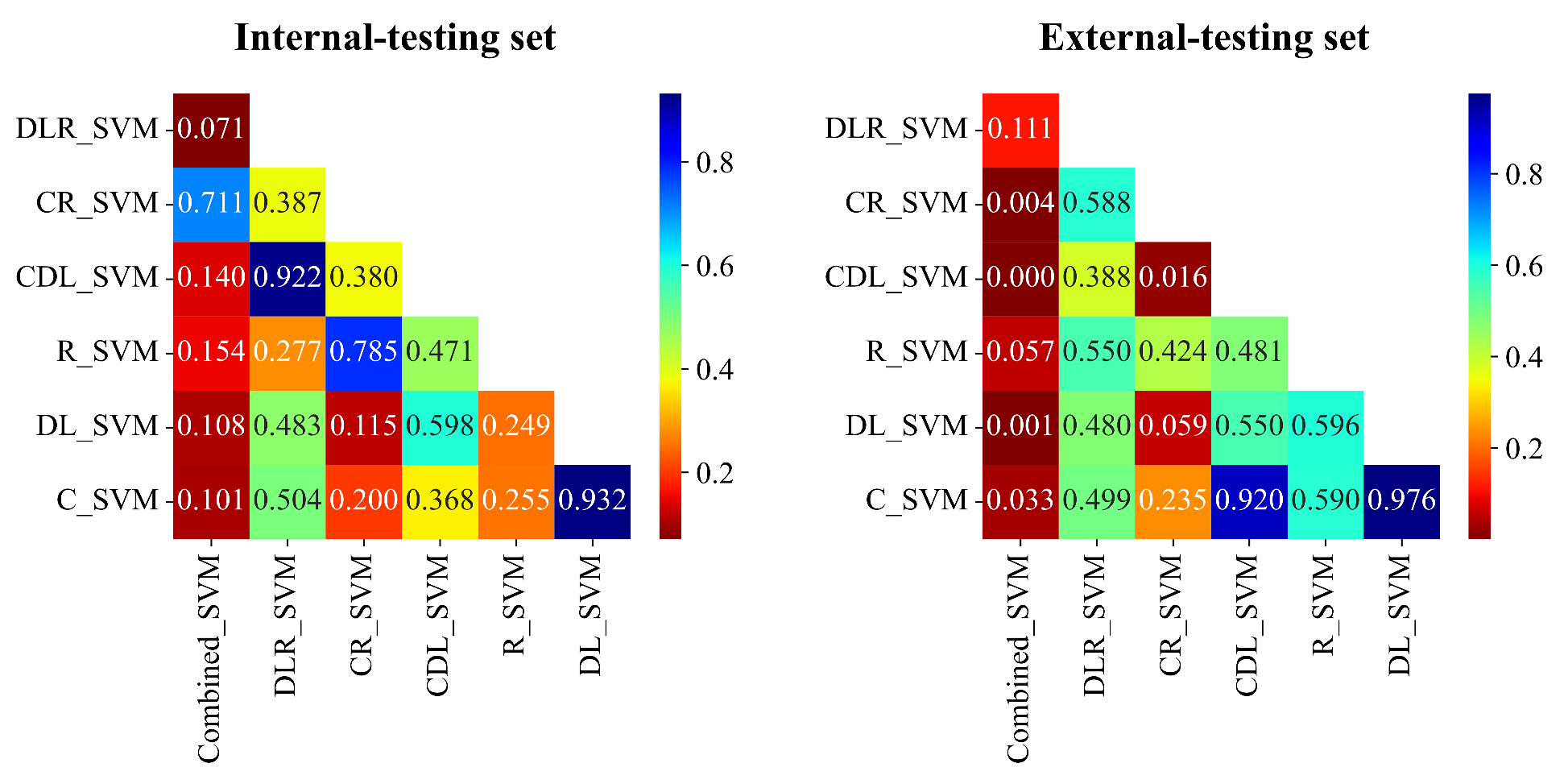


Supplementary Fig.4 Comparison of different models through the Delong test.

Supplementary Table 1: Data Collection Periods by Institution.

| Institution | Full Name | USC Collection Period | EEC Collection Period |
| --- | --- | --- | --- |
| Institution I | Shantou Central Hospital | January 2017 – October 2023 | March 2023 – October 2023 |
| Institution II | Sun Yat-Sen Memorial Hospital | May 2017 – June 2023 | March 2023 – October 2023 |
| Institution III | Sun Yat-Sen University Cancer Center | August 2018 – July 2023 | March 2023 – October 2023 |
| Institution IV | Cancer Hospital of Shantou University Medical College | March 2020 – August 2023 | March 2023 – October 2023 |

Supplementary Table 2: Detailed Sequences Scanning Parameters in Four MR Scanners.

| MRI scanner | Image type | Sequence | Acquisition plane | TR/TE (ms) | FOV (cm) | Section thickness(mm) | b-Value(s/mm2) |
| --- | --- | --- | --- | --- | --- | --- | --- |
| 3.0-T | T2WI | FSE | Sagittal, Axial | 4000-5000/85 | 26-38 | 4 | - |
| Siemens Verio | DWI | EPI | Axial | 4500/93 | 26 | 6 | 0,1000 |
| (Institution I) | CE-T1WI | VIBE | Sagittal, Axial | 3.92/1.39 | 30-38 | 3 | - |
| 1.5-T | T2WI | FSE | Sagittal, Axial | 3900-5610/81-91 | 32-36 | 4 | - |
| Siemens Area | DWI | EPI | Axial | 4500/82 | 23 | 4 | 0,1000 |
| (Institution II) | CE-T1WI | VIBE | Sagittal, Axial | 3.23/1.21 | 38 | 1.2 | - |
| 1.5-T | T2WI | FSE | Sagittal, Axial | 3920-7660/144-147 | 18-30 | 3-4 | - |
| Siemens Avanto | DWI | EPI | Axial | 3000/91 | 38 | 5 | 0,1000 |
| (Institution III) | CE-T1WI | 3D-GRE | Sagittal, Axial | 5.43/2.5 | 25 | 1.6 | - |
| 3.0-T | T2WI | FSE | Sagittal, Axial | 4220-5500/102-125.4 | 22-30 | 3-4 | - |
| GE Discovery HD750 | DWI | EPI | Axial | 4000/56.1 | 34 | 5 | 0,800 |
| (Institution IV) | CE-T1WI | 3D LAVA-XV | Sagittal, Axial | 7.9/4.1 | 35 | 1 | - |

*T2WI* = T2-weighted imaging, *DWI* = diffusion-weighted imaging, *DCE* = dynamic contrast-enhancement, *CE-T1WI* = contrast enhanced T1-weighted imaging, *FSE* = fast spin echo, *EPI* = echo planar imaging, *VIBE* = volumetric interpolated breath-hold examination, *LAVA-XV* = liver acquisition with volume acceleration-extended volume, *3D-GRE* = three-dimensional gradient-echo, *TE* = echo time, *TR* = repetition time, *FOV* = field of view.

Supplementary Table 3: Inter-observer agreement for clinical-radiological characteristics

| **Category** | **Feature** | **Statistical Method** | **Value** |  |
| --- | --- | --- | --- | --- |
| **Morphological Features** | Tumor Size (max diameter) | ICC | 0.92 |  |
|  | Tumor Volume | ICC | 0.90 |  |
|  | Endometrial Thickness (ET) | ICC | 0.89 |  |
|  | ET/AP Ratio | ICC | 0.88 |  |
|  | Tumor Border (Well-defined vs Ill-defined) | Cohen's κ | 0.92 |  |
|  | Growth Pattern (Expansile vs Infiltrative) | Cohen's κ | 0.89 |  |
|  | Diffuse Distribution (Absent vs Present) | Cohen's κ | 0.92 |  |
|  | Presence of Necrosis (Absent vs Present) | Cohen's κ | 0.93 |  |
|  | Presence of Hemorrhage (Absent vs Present) | Cohen's κ | 0.98 |  |
| **Signal Intensity & Enhancement** | SIR on T2WI | ICC | 0.89 |  |
|  | SIR on DWI | ICC | 0.93 |  |
|  | SIR on CE-T1WI | ICC | 0.87 |  |
|  | Inhomogeneity on T2WI (Homogeneous vs Heterogeneous) | Cohen's κ | 0.87 |  |
|  | Enhancement Pattern (Homogeneous vs Heterogeneous) | Cohen's κ | 0.83 |  |
| **Tumor Invasion & Spread** | Deep Myometrial Invasion (<50% vs ≥50%) | Cohen's κ | 0.81 |  |
|  | Cervical Stromal Invasion (Absent vs Present) | Cohen's κ | 0.84 |  |
|  | Adnexal Involvement (Absent vs Present) | Cohen's κ | 0.89 |  |
|  | Pelvic Lymph Node Metastasis (Absent vs Present) | Cohen's κ | 0.79 |  |
|  | Peritoneal Dissemination (Absent vs Present) | Cohen's κ | 1.00 |  |
|  | Abnormal Ascites (Absent vs Present) | Cohen's κ | 1.00 |  |
